# Telomere dysfunction is associated with exacerbated intermittent hypoxia-induced cognitive deficits and nerve damage

**DOI:** 10.3389/fnagi.2026.1758688

**Published:** 2026-03-06

**Authors:** Ying Guo, Yuyang Miao, Jin Tan, Rui Zhao, Duo Jiang, Minghui Zou, Feng Wang, Qiang Zhang

**Affiliations:** 1 Department of Geriatrics, Tianjin Key Laboratory of Elderly Health, Tianjin Geriatrics Institute, Tianjin Medical University General Hospital, Tianjin, China; 2 Key Laboratory of Post-Neuroinjury Neuro-Repair and Regeneration in the Central Nervous System, Ministry of Education, State Key Laboratory of Experimental Hematology, Tianjin, China; 3 Tianjin Academy of Traditional Chinese Medicine Affiliated Hospital, Tianjin, China; 4 Department of Genetics, Tianjin Medical University, Tianjin, China; 5 Department of Endocrinology and Metabolism, Tianjin Medical University General Hospital, Tianjin, China; 6 The Province and Ministry Co-sponsored Collaborative Innovation Center for Medical Epigenetics, Institute of Prosthodontics School and Hospital of Stomatology, Tianjin Medical University, Tianjin, China

**Keywords:** cellular senescence, cognitive decline, intermittent hypoxia, neuronal susceptibility, obstructive sleep apnea, senolytics

## Abstract

Cognitive impairment associated with obstructive sleep apnea (OSA) is more prevalent and severe in the elderly, possibly due to age-related increases in neuronal susceptibility to intermittent hypoxia (IH). As telomere dysfunction is a key driver of cellular aging, this study aimed to characterize the interaction between telomere dysfunction and IH, and to explore the associated molecular alterations. Using telomere-damaged PC12 cells and G3 Tert^−/−^ progeria mice exposed to IH, we assessed cellular stress responses, apoptosis, cognitive function, and hippocampal structural changes. The effects of the senolytic agent fisetin (*in vivo*) and the mTOR inhibitor rapamycin (*in vitro*) were evaluated. Transcriptomic analysis was performed on cells. IH-exposed G3 Tert^−/−^ mice displayed exacerbated cognitive deficits and hippocampal atrophy compared to wild-type controls, which were significantly ameliorated by fisetin treatment (vs. IH-G3 Tert^−/−^: cognitive deficit, *p* = 0.028; hippocampal atrophy, *p* < 0.01). Correspondingly, telomere-damaged PC12 cells exhibited a heightened stress response to IH, manifested by increased p21, SA-*β*-gal and apoptosis upon IH, an effect also mitigated by rapamycin. RNA sequencing of these cells revealed a distinct inflammatory signature under IH, with enrichment in pathways like TNF and IL-17 signaling and identification of IL-6, CXCL10, and ICAM1 as key hub genes. Our findings indicate that telomere dysfunction is associated with exacerbated IH-induced cognitive deficits and nerve damage. We identify a corresponding inflammatory transcriptomic signature and provide preliminary evidence that interventions targeting these senescence-associated pathways can confer protection. This provides a new mechanistic perspective on aging-related susceptibility and outlines a translational roadmap for future investigation into OSA-related cognitive decline.

## Introduction

Obstructive sleep apnea (OSA) is a common sleep-related breathing disorder characterized by recurrent upper airway obstruction during sleep, leading to intermittent hypoxia (IH), sleep fragmentation, and excessive daytime sleepiness (Deegan and McNicholas, 1995; Levy et al., 2015; McNicholas and Pevernagie, 2022). Global epidemiological data estimate that approximately 936 million adults aged 30 to 69 years, across both sexes, have mild to severe OSA (Benjafield et al., 2019). Beyond its association with increased mortality, OSA contributes to a spectrum of serious health conditions, including cardiovascular disease, metabolic syndrome, diabetes, and cognitive impairment and dementia, which are of growing concern (Drager et al., 2013; Gileles-Hillel et al., 2016; Yeghiazarians et al., 2021; Chang et al., 2023; Huang et al., 2025). Clinical observations reveal that up to 26% of OSA patients exhibit cognitive deficits, and a 2024 meta-analysis has further quantified this association, demonstrating that OSA increases the risk of cognitive impairment and all-cause dementia by more than 50% (Leng et al., 2017; Tian et al., 2024). Both IH and sleep fragmentation (SF), the primary pathophysiological features of OSA, contribute to the induction and exacerbation of cognitive impairment (Yaffe et al., 2014). Extensive clinical, animal, and cellular studies have demonstrated that IH can induce hippocampal neuronal apoptosis by triggering oxidative stress and inflammatory responses, which represents the principal mechanism underlying OSA-related cognitive dysfunction (Lal et al., 2012; Zhang et al., 2013). Furthermore, among older adults, the prevalence of mild cognitive impairment or dementia in OSA patients rises significantly, reaching as high as 44.8%, suggesting that aging may confer heightened susceptibility to cognitive decline in the context of OSA (Ayalon et al., 2010; Bombois et al., 2010; Yaffe et al., 2011).

While the severity of OSA is a known contributor to cognitive dysfunction, other factors such as genetic predisposition (e.g., APOE ε4 allele), history of stroke, and traumatic brain injury (TBI) also exacerbate the risk of neurocognitive impairment, particularly in older adults (Gozal et al., 2007; Wilde et al., 2007; Cosentino et al., 2008; Castriotta and Murthy, 2011). Beyond these distinct disease-related factors, the physiological process of aging itself is considered a foundational state that increases the susceptibility of multiple tissues to various insults. For example, glomerular podocytes in aged mice display greater sensitivity to oxidative stress compared to their younger counterparts, contributing to age-related renal dysfunction (Hartleben et al., 2010). Similarly, increased susceptibility of aging nerve cells to oxidative damage, along with a decline in antioxidant defenses, is implicated in the pathogenesis of neurodegenerative disorders such as Alzheimer’s disease (AD) (Driver et al., 2000; Venkateshappa et al., 2012; Salminen and Paul, 2014; Wang et al., 2022). However, the specific cellular and molecular mechanisms driving this age-associated susceptibility, particularly the role played by telomere dysfunction, remain poorly understood. A central yet underexplored hypothesis is that telomere dysfunction may specifically heighten neuronal susceptibility to IH-induced damage.

In this study, we employed a model of accelerated telomere dysfunction (G3 Tert^−/−^ mice) and telomere-damaged PC12 cells to investigate the interaction between telomere dysfunction and IH. Given that IH is the central pathological insult in OSA and neuronal apoptosis is a key mechanism of OSA-related cognitive decline, we exposed both models to IH. Behavioral testing, magnetic resonance imaging (MRI), cellular stress markers, apoptosis and RNA sequencing were conducted. Furthermore, to explore a potential therapeutic avenue, we evaluated the effects of the senolytic agent fisetin in our *in vivo* model. This study aimed to characterize the relationship between telomere dysfunction and increased neuronal susceptibility to IH-induced damage, preliminarily explore the molecular features underlying this association using RNA sequencing, and investigate whether targeting senescence-related processes can mitigate the associated pathological outcomes.

## Materials and methods

### Cell culture and IH exposure

PC12 cells were purchased from the China Infrastructure of Cell Line Resource (Beijing, China). Cells were cultured in DMEM high glucose mediumsupplemented with 10% fetal bovine serum (FBS) and 1% penicillin/streptomycin at 37 °C in a chambercontaining 5% CO₂. PC12 cells in rapamycin group were treated with 20 nM rapamycin (Abcam, UK) before IH exposure. The IH cell model was established following our previously published protocol (Guo et al., 2025). Prior to IH exposure, the culture medium was replaced with fresh serum-free medium. PC12 cells were transferred to a Plexiglas chamber, which was cyclically flushed with hypoxic gas mixture (1.5% O₂, 5% CO₂, N₂; 600 s per cycle) or normoxic gas mixture (21% O₂, 5% CO₂, N₂; 300 s per cycle) for a total of 12 h. The chamber was regulated to maintain optimal conditions at 37 °C with 45% humidity under relatively germ-free conditions.

### Preparation of Cas9 sgRNA telomere targeting (Cas tel) virus

The Cas tel and Cas scramble plasmids were generously provided by Professor Yong Zhao from Sun Yat-sen University. A mixture containing 7.5 μg of pSPAX, 2.5 μg of pMD2. G, and 10 μg of Cas Tel plasmid was prepared and incubated for 20 min. Subsequently, the mixture was added to HEK293T cells cultured in Opti-MEM (Gibco, USA). After 6–8 h, the medium was replaced with fresh DMEM medium. The supernatant was collected after 48 or 72 h of culture and subjected to ultracentrifugation (4 °C, 25,000 rpm, 2 h) for viral concentration.

### Cell infection

PC12 cells were seeded in 6-well plates at a density of 3 × 10^5^ per well. Fresh medium was replaced the next day, and 1 mL of virus was added. After 24 h, the medium was replaced with fresh medium again, followed by antibiotic selection with puromycin (2 μg/mL) after another 24 h.

### Construction of G3 Tert−/− mice

Telomerase reverse transcriptase (Tert) maintains genomic stability by preserving telomere length through its activity. Building on our previous work, telomerase deficient mice was established (Zhao et al., 2023). Briefly, Tert^+/−^ mice (generously provided by Professor Yusheng Cong) were crossed to obtain Tert^−/−^ mice. We successfully established G1 Tert^−/−^ and G3 Tert^−/−^ mice, and selected female G3 Tert^−/−^ mice as a telomerase-deficient progeria mouse model for our experiment. Mice were kept under specific pathogen-free (SPF) conditions with free access to sterilized food and water. All animal experiments were approved by the Animal Ethics Committee of Tianjin Medical University General Hospital (IRB2025-DWFL-050).

### IH exposure (mice)

IH was induced as previously described (Zhang et al., 2023; Sun et al., 2026). At 6–8 weeks of age, mice in each group were subjected to IH treatment for 9 weeks, 8 h per day (from 9:00 a.m. to 5:00 p.m.). The IH regimen was administered in specialized Plexiglas chambers, with each 90-s cycle consisting of a 55-s hypoxic phase (FiO_2_ rapidly reduced to 5% via nitrogen infusion) followed by a 35-s re-oxygenation phase (FiO_2_ restored to 21% using compressed air). This cycle was repeated 320 times per day. Throughout the exposure, chamber temperature and humidity were maintained at 22–24 °C and 45%, respectively. Normoxic mice were housed in identical chambers under a constant FiO_2_ of 21% for the same duration. All mice were returned to standard cage housing with room air for the remaining 16 h each day.

### Experimental design and treatment

To evaluate whether a telomere dysfunction-driven progeroid phenotype exacerbates neuronal susceptibility to IH, mice were allocated into five groups: wild-type (WT), G3 Tert^−/−^, IH-exposed WT (IH), IH-exposed G3 Tert^−^/^−^ (IH-G3 Tert^−^/^−^), and IH-exposed G3 Tert^−^/^−^ treated with the senolytic agent fisetin (Fisetin). Beginning at 8 weeks of age, mice in fisetin group received gastric gavage with fisetin (20 mg/kg, Meilunbio) six times per week for a total of 9 weeks. Before conducting the gavage procedure, animals were fasted for 6–8 h. WT, G3 Tert^−/−^, IH, and IH-G3 Tert^−/−^ groups received an equivalent volume of placebo. Fisetin was prepared according to the instructions by dissolving in 10% ethanol, 30% PEG 400, and 60% Phosal 50 pg. before gavage.

### Barnes maze test

The Barnes maze test was conducted during the 9th week of IH exposure to assess spatial learning and memory. Prior to formal testing, mice were habituated to the experimental environment. This was followed by four consecutive days of training, a two-day rest period, and finally a probe trial on day seven with the escape tunnel removed. Mice were allowed to freely explore the arena for 2 min while their behavior was videotaped for analysis. The primary outcomes were escape distance and latency, as well as the number of successful crossings over the target hole. These parameters were automatically acquired and analyzed using Digbehv Animal Behavior Analysis System, with manual verification of tracking accuracy to ensure data fidelity.

### *In vivo* brain magnetic resonance imaging (MRI)

We performed mice brain MRI acquisition on a 9.4 T MRI scanner (Bruker BioSpec 94/30 USR, Germany). Isoflurane (5%) was used to anesthetize mice and 1–2% isoflurane was used for maintenance. For structural MRI of the brain, we first acquired 2D T2-weighted TurboRARE images using the following parameters: repetition time (TR) = 3,205 ms, echo time (TE) = 33 ms, 20 contiguous slices with 1 mm thickness, field of view (FOV) = 15 mm × 15 mm. The 3D T2-weighted TurboRARE sequence was acquired using the following parameters: TR = 1800 ms, TE = 37 ms, RARE factor = 12, FOV = 18 mm × 18 mm × 9 mm, 60 contiguous slices with 0.15 mm thickness (Cheng et al., 2023).

All subsequent analyses were performed under strict blinding conditions. For hippocampal volume analysis, the original DICOM files were first converted to NIFTI format using dcmniix, followed by 3-fold voxel size augmentation through DPABI. DICOM files were anonymized by an independent researcher not involved in morphometry, using random numerical IDs. Hippocampus was then automatic segmented using SPM12 software with the Turone Mouse Brain Atlas and Template (TMBTA) as the reference space. The TMBTA delineated seven distinct hippocampal subregions: hippocampal formation, CA1, CA2, and CA3 regions, along with three dentate gyrus (DG) subdivisions - polymorph layer, molecular layer, and granule cell layer of DG. The volumes of all subregions were summed to represent total hippocampus. To exclude differences in intracranial size, total intracranial volume (TIV) was calculated as the concomitant variable for normalization of hippocampal volume.

Finally, for brain T2 map imaging, the multiple gradient-echo sequence was acquired with the following parameters: TR = 850 ms, TE = 4 ms, flip angle = 25°, FOV = 18 mm × 18 mm, 18 contiguous slices with 0.141 mm thickness. A researcher blinded manually defined the hippocampus as a region of interest (ROI) in all MR imaging slices of each mouse. The mean T2 relaxation time of hippocampal slices was calculated for each mouse and used as the final quantitativemeasurement.

### Telomere dysfunction-induced foci (TIFs) assay

PC12 cells were cultured on coverslips, fixed with 4% paraformaldehyde (PFA) for 15 min at room temperature, and permeabilized with 0.2% Triton X-100 for 20 min. After 2 × 5 min washes with phosphate-buffered saline (PBS), The cells were dehydrated through a graded ethanol series before being air-dried at room temperature. For telomere fluorescence *in situ* hybridization (FISH), 0.6 μL of TelC-488-labeled telomere probe (Panagene, Korea) was combined with 50 μL of hybridization buffer containing 35 μL of 70% formamide, 0.5 μL Tris–HCl pH 7.2, 5 μL of 1 × blocking solution, and 9 μL distilled water. The cells were co-incubated with the hybridization buffer. Slides were then denatured at 80 °C for 3 min using a heating block and subsequently hybridized overnight at room temperature in a humidity box. Slides were sequentially washed 2 × 15 min in solutioncontaining 70% formamide, 0.1 M Tris–HCl pH 7.2, 10% BSA and distilled water, followed by 3 × 5 min washes in TBST (1 × TBS, 1% TWEEN-20) and blocked with PBG buffer (1 × PBS, 0.5% BSA, 1% gelatin) for 1 h. Cells were co-incubated with rabbit anti-53BP1 antibody (1:1000 dilution, abcam, ab175933, GR3310237-5) diluted with PBG for 1 h at room temperature. Following 4 × 5 min washes with PBST, cells were co-incubatedwith a fluorophore-conjugated secondary antibody (1:2000 dilution, Thermo Fisher Scientific, A11035, 2,701,068) for 30 min at room temperature. Then cells were dehydrated with a graded ethanol series after 4 × 5 min PBST washes. Nuclei were stained with DAPI (Abcam, UK). Fluorescent images were obtained using a Fluorescence microscope (Zeiss, Germany) and Zen software. For each experimental condition, three independent cell culture and treatment experiments were performed. In each experiment, images were acquired from three randomly selected, non-overlapping fields of view using a 100x oil immersion objective. TIFs were quantified using ImageJ software by counting all foci within each cell that displayed an intact nucleus.

### Senescence-associated β-galactosidase (SA-β-gal) staining of cells

Cellular senescence in PC12 cells was assessed using a Senescence-associated β-Galactosidase Staining Kit (Beyotime, C0602, China), following the manufacturer’s protocol. Cells were fixed with 4% PFA for 10 min. After 3 × 5 min PBS washes, cells were incubated with the SA-β-gal staining working solution (pH 6.0) in a dry, CO_2_-free incubator at 37 °C for 12 h in the dark. Following incubation, cells were observed under an optical microscope. For each sample, at least three random, non-overlapping fields were captured. SA-β-gal-positive cells (displaying blue staining) and the total cell number were manually counted using ImageJ software. Each experiment was independently repeated three times.

### Immunofluorescence staining for p21

PC12 cells were cultured on coverslips, fixed with 4% PFA for 15 min at room temperature. After washing with PBS, cells were permeabilized and blocked simultaneously by incubating in PBS containing 0.3% Triton X-100 and 3% bovine serum albumin (BSA) for 2 h at room temperature. Cells were incubated with rabbit anti-p21 antibody (1:200 dilution, Proteintech, 28,248-1-AP, 00137197) diluted with PBS overnight at 4 °C. After washing, cells were incubated with a fluorophore-conjugated secondary antibody (1:200 dilution, Thermo Fisher Scientific, A21206, 2,156,521) for 1 h at room temperature in the dark. DAPI (Abcam, UK) was used for staining the nucleus. After incubation, cells were observed under a fluorescence microscope. For each sample, at least three random, non-overlapping fields were captured. P21-positive cells (displaying green staining) and the total cell number were manually counted using ImageJ software. Each experiment was independently repeated at least three times.

### DNA damage assay

DNA damage in PC12 cells was assessed using the DNA Damage Assay Kit (Beyotime, C2035S, China). Briefly, samples were first incubated with the kit’s primary antibody against *γ*-H2AX, followed by incubation with a fluorescence-labeled secondary antibody. After incubation, cells were observed under a fluorescence microscope. For each sample, at least three random, non-overlapping fields were captured. γ-H2AX-positive cells (displaying green staining) and the total cell number were manually counted using ImageJ software. Each experiment was independently repeated three times.

### TdT-mediated dUTP nick-end labeling (TUNEL)

PC12 cells were cultured on glass slides, washed three times withPBS, and fixed with 4% PFA for 15 min at room temperature for subsequent staining. Mice were transcardially perfused with ice-cold PBS followed by 4% PFA for tissue fixation. Whole brains were carefully extracted and fixed with 4% PFA at 4 °C for 24 h. The brain samples were then embedded in optimal cutting temperature (OCT, Sakura, USA) after graded sucrose dehydration. The brain samples were sectioned at appropriate thickness using a frozen slicer maintained at −20 °C. The cells or tissue sections were placed at room temperature for 15 min following removal from −20 °Crefrigerator before staining. Following 3 × 5 min PBS washes, cells or brain tissue sections were permeabilized with 0.3% Triton X-100 for 30 min. A TdTdUTP nick-end labeling (TUNEL) apoptosis assay kit (Beyotime Biotechnology Co., Ltd., China) was used to assess cell apoptosis. Cells or brain tissue sections were incubated with TUNEL reaction mixture for 1 h at 37 °C in the dark, followed by nuclear staining with DAPI (Abcam, UK). Fluorescent images were obtained using a Fluorescence microscope (Zeiss, Germany) and Zen software. For *in vitro* experiments, three independent biological replicates were performed per condition. From each replicate, three random, non-overlapping fields of view were analyzed. For *in vivo* experiments, each group comprised n = 5 mice. From each mouse, three consecutive brain tissue sections were collected, and three non-overlapping fields of view were analyzed per section.

### RNA extraction and sequencing (RNA-Seq)

Total RNA was isolated from PC12 cells in four groups using Trizol (Thermo Fisher Scientific, US), including NC group, Cas tel group, IH 12 h group and IH 12 h-Cas tel group. Three samples are contained in each group. RNA integrity and quantity were assessed using Agilent 2,100 bioanalyzer. After library preparation and quality assessment, RNA sequencing was conducted on the Illumina platform. Following quality control processing, high-quality clean reads were mapped to the reference genome. StringTie was then performed to assemble the mapped reads of each sample.

Quantification of gene expression level was calculated using featureCounts. Differential gene expression analysis was conducted using DESeq2, which implements a negative binomial distribution-based statistical model for RNA-seq data analysis. Statistical significance was adjusted using the false discovery rate (FDR) method for multiple testing, with an adjusted significance threshold of Q-value≤0.05.

### Statistical analysis

Data are presented as the mean ± standard deviation (SD). The Shapiro–Wilk test was used to assess the normality of the data. Statistical analysis was performed using GraphPad Prism v7.0. Comparisons between two independent groups were performed using the unpaired, two-tailed Student’s t-test. For comparisons across multiple groups, a one-way analysis of variance (ANOVA) was performed. When the overall ANOVA was significant (*p* < 0.05), Tukey’s honestly significant difference (HSD) *post hoc* test was used for all pairwise comparisons between groups. A *p*-value of < 0.05 was considered statistically significant for all tests.

## Results

### G3 Tert^−^/^−^ mice showed exacerbated cognitive deficits under IH

To investigate the interaction between telomere dysfunction and IH *in vivo*, we utilized third-generation telomerase reverse transcriptase knockout mice (G3 Tert^−^/^−^), a well-established progeria model characterized by critically short telomeres and accelerated aging phenotypes. An OSA model was then established by exposing mice to IH, followed by cognitive assessment using the Barnes maze test conducted 9 weeks after IH exposure.

**Figure 1:**
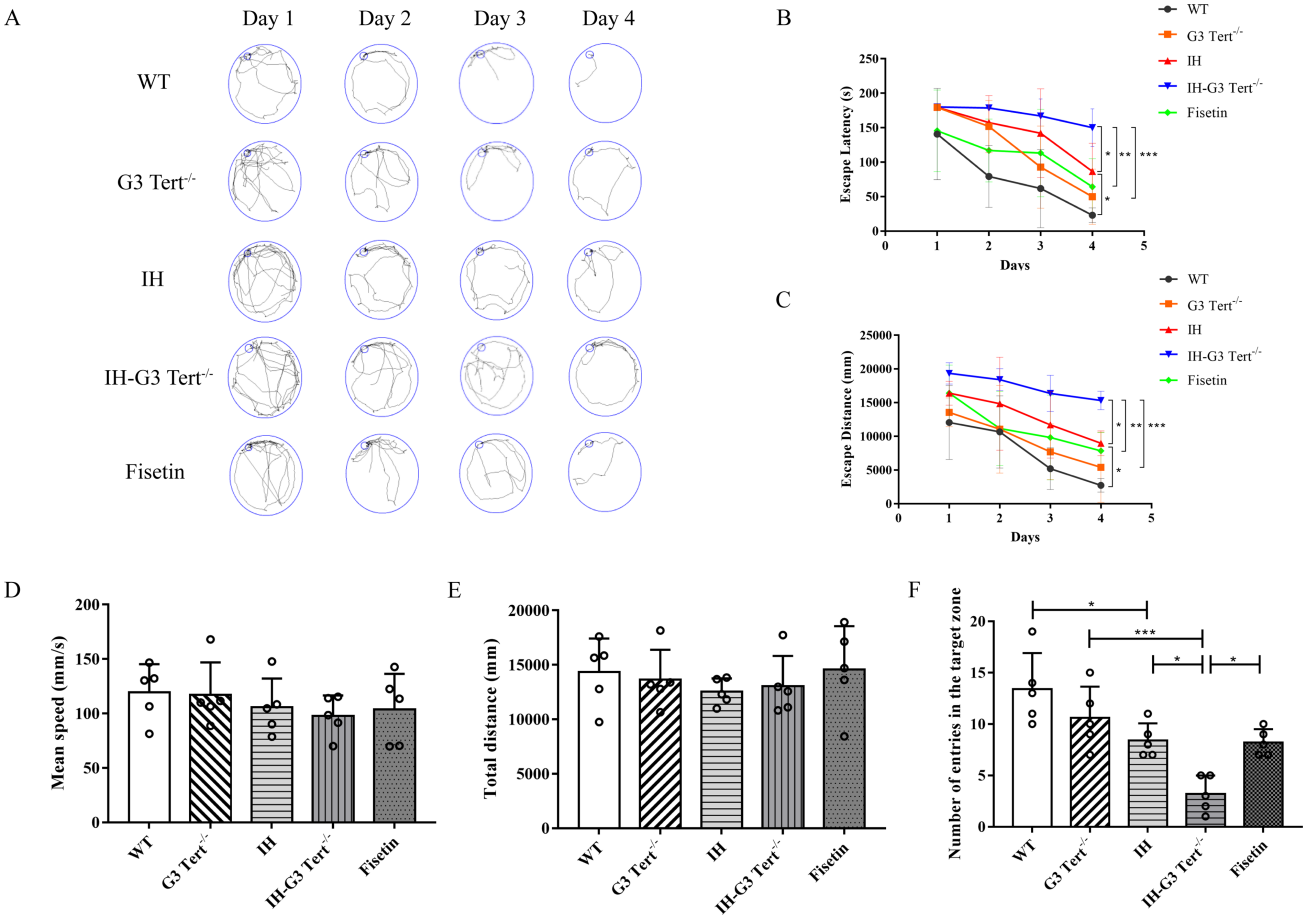
G3 Tert^−/−^ mice showed exacerbated cognitive deficits under IH. **(A)** The training paths of mice in the five groups of Barnes Maze. **(B,C)** Escape latency and distance recorded for each training day (*n* = 5). **(D,E)** The mean speed and total distance in the probe phase of Barnes Maze (*n* = 5). **(F)** The number of escape tunnel crossings during the probe trial of Barnes Maze (*n* = 5). *p < 0.05, ***p < 0.001.

During the training phase (days 1–4) of the Barnes maze, escape latency and escape distance progressively decreased in all groups—including WT, G3 Tert^−^/^−^, IH, and IH–G3 Tert^−^/^−^ mice—indicating learning acquisition. However, on day 4, both escape latency and distance in IHmice increased approximately fourfold compared to those in the WT group. Moreover, IH-G3 Tert^−/−^ mice exhibited further impairments, with approximately 70% longer escape latency and distance than IH mice. Notably, treatment with the senolytic agent fisetin significantly improved the performance of IH–G3 Tert^−^/^−^ mice in these parameters (Figures 1A–C).

On day 7, during the probe trial in which the escape tunnel was removed, there were no significant differences among the groups in total distance traveled or average movement speed (Figures 1D,E), suggesting that IH treatment did not impair general motor function. However, IH mice crossed the former location of the escape tunnel significantly fewer times than WT mice, indicating impaired spatial memory (Figure 1F). These results suggest that the prolonged escape latency, increased escape distance, and reduced tunnel crossings in IH mice stem from deficits in spatial learning and memory rather than motor impairment. Notably, IH–G3 Tert^−^/^−^ mice displayed more severe cognitive deficits compared to IH mice, and was partially rescued by fisetin treatment (Figure 1F). Together, these results demonstrate that G3 Tert^−^/^−^ mice display exacerbated cognitive deficits following IH exposure, and that these deficits are ameliorated by fisetin treatment.

### G3 Tert^−^/^−^ mice developed more severe hippocampal damage under IH

Magnetic resonance imaging (MRI) is a noninvasive and highly informative modality that enables multidimensional structural and functional assessment of the brain. Clinically, it plays a critical role in evaluating cerebral integrity and diagnosing neurological disorders. Given the well-established link between hippocampal atrophy and mild cognitive impairment (MCI), structural MRI (sMRI) was performed immediately after the Barnes maze test. Three-dimensional T2-weighted TurboRARE sequences were acquired to assess brain structure, followed by volumetric quantification of the hippocampus and its subregions.

**Figure 2:**
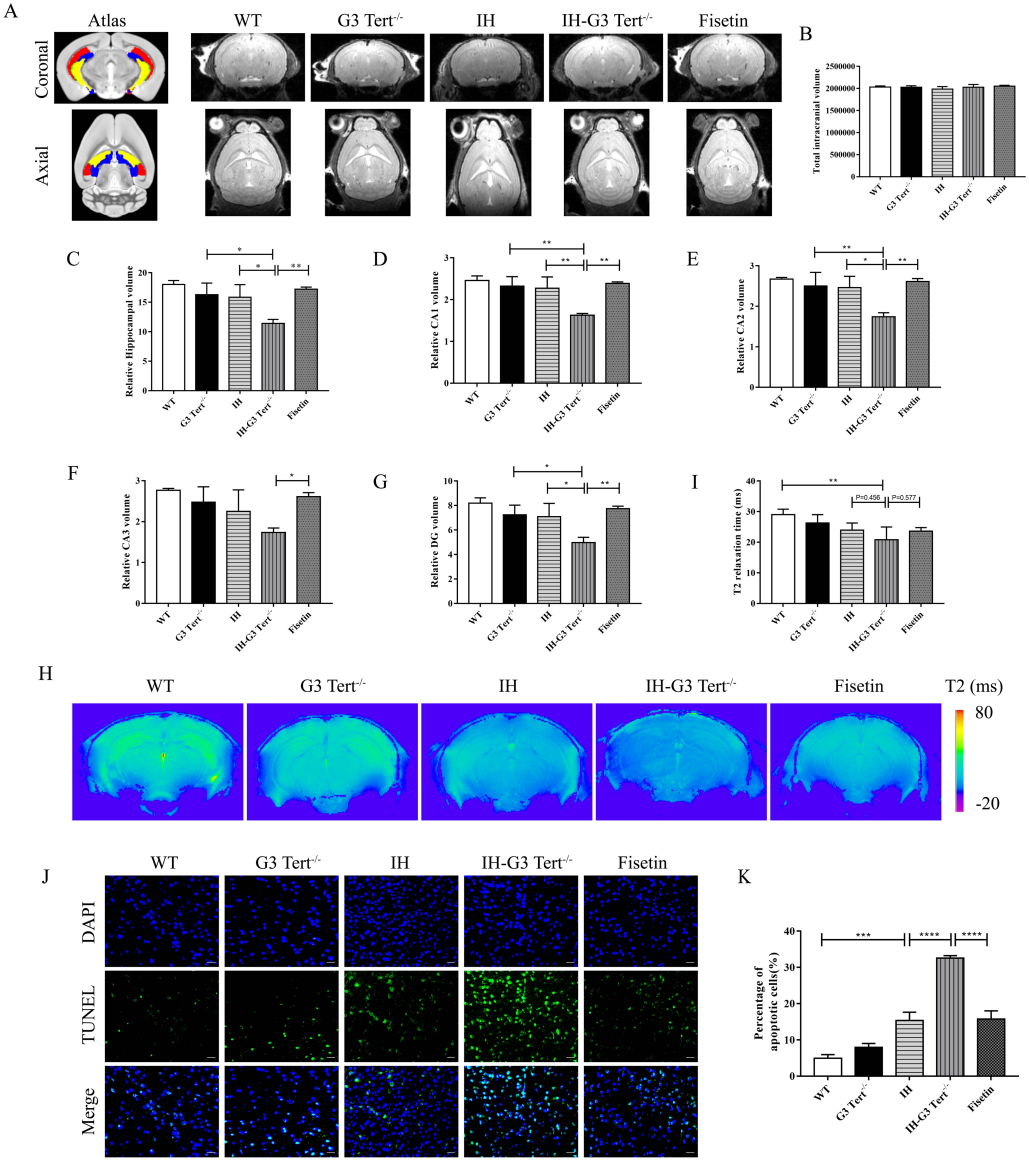
G3 Tert^−/−^ mice developed more severe hippocampal damage under IH. **(A)** Coronal and axial atlas of mouse brain anatomical template annotated with major hippocampal subregions (Red: CA1; Green: CA2; Yellow: CA3; Blue: DG) and representative T2-weighted MRI images of mouse brain. **(B–G)** Volumetric analysis of the intracranial, hippocampus and its subregions (*n* = 5). **(H)** Representative T2 map images. **(I)** T2 relaxation time measured in hippocampus (*n* = 5). **(J, K)** Cell apoptosis of brain tissue sections was assessed by TUNEL (*n* = 3). Scale bars: 200 μm. *p < 0.05, **p < 0.01, ***p < 0.001, ****p < 0.0001.

Total intracranial volume (TIV) did not significantly differ among groups, excluding intracranial size as a confounding factor (Figures 2A,B). A significant reduction in total hippocampal volume was observed in IH–G3 Tert^−^/^−^ mice compared to IH mice. This volume loss was particularly pronounced in specific subregions, including CA1, CA2, and the dentate gyrus (DG). Notably, treatment with fisetin significantly attenuated hippocampal volume loss in IH–G3 Tert^−^/^−^ mice (Figures 2C–G).

Importantly, alterations in brain microstructure often precede overt volumetric atrophy and can be detected via intrinsic MRI parameters. Among these, T2 relaxation time is a sensitive marker for microstructural changes due to pathological processes. Previous studies have demonstrated that reduced T2 values are correlated with the progression of AD, but so far no research has detected a significant association between OSA models and reduced brain T2 values. To further assess microstructural integrity, T2 mapping MRI was performed using a multi-gradient-echo (MGE) sequence. Axial T2 map images revealed reduced hippocampal T2 signal intensity in IH-G3 Tert^−/−^ mice compared to IH mice, an effect that was mitigated by fisetin treatment (Figure 2I). Quantitative analysis confirmed that fisetin treatment significantly attenuated the reduction in hippocampal T2 values in IH-G3 Tert^−/−^ mice (*p* = 0.577 vs. untreated IH-G3 Tert^−/−^ mice; *p* = 0.456 vs. IH mice) (Figure 2H).

To evaluate neuronal death, TUNEL staining was conducted. The number of apoptotic cells in the hippocampus was significantly elevated in IH mice, and even more pronounced in IH–G3 Tert^−^/^−^ mice. This increase in apoptosis was attenuated by fisetin treatment (Figures 2J,K). Collectively, these results demonstrate that G3 Tert^−/−^ mice showed exacerbated hippocampal atrophy, microstructural disruption, and cellular apoptosis following IH exposure, all of which were alleviated by fisetin treatment.

### Telomere-damaged PC12 cells displayed heightened stress responses to IH

**Figure 3:**
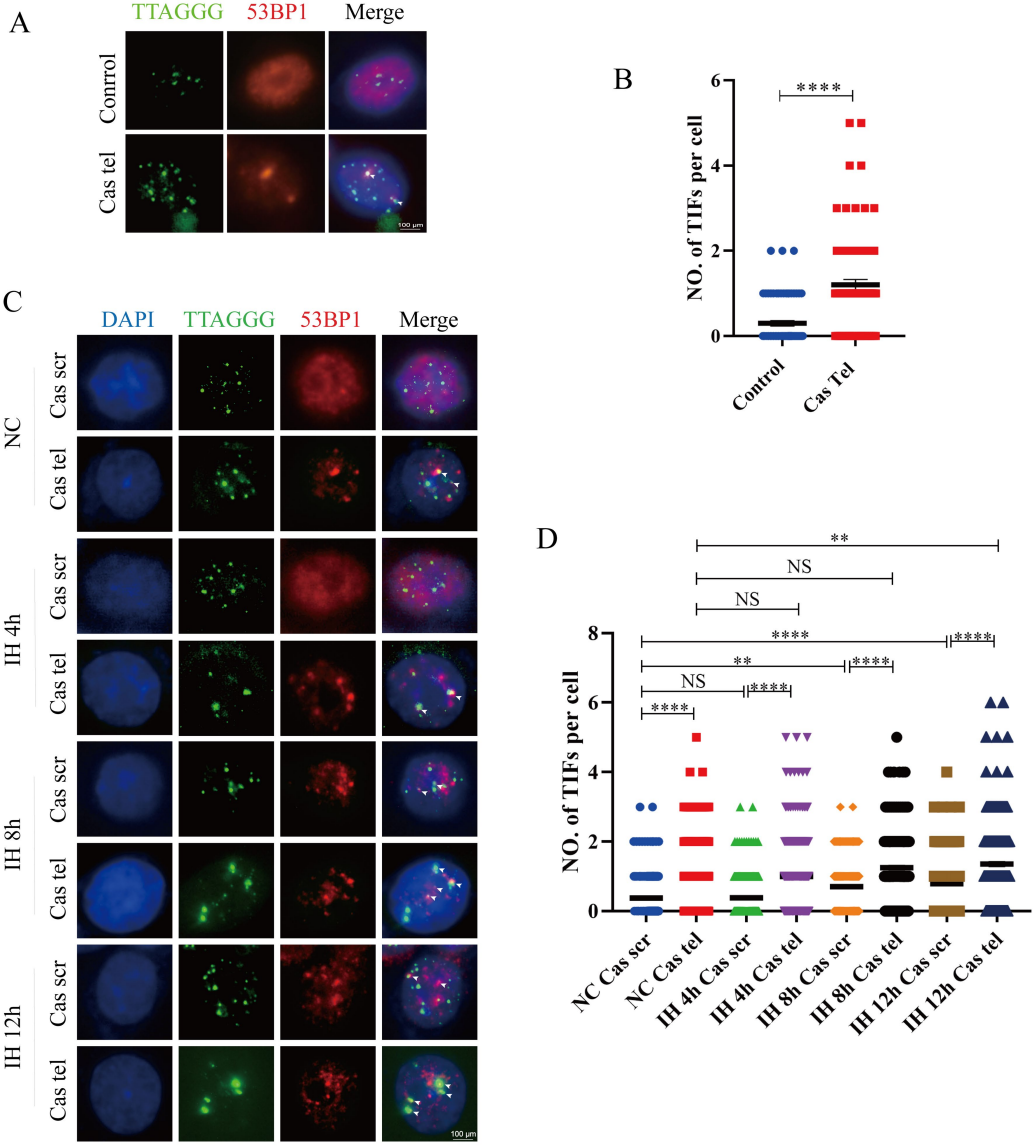
Construction of telomere-damaged PC12 cells model. **(A)** IF images showed TIF formation in control and Cas tel groups. TIFs were assigned if there is co-localization of 53BP1 (red) and Telc-488 labeled telomere end (green). Scale bars: 100 μm. **(B)** The number of TIFs per cell are counted and plotted. Scale bars: 100 μm. **(C)** IF images showed TIF formation in control and Cas tel groups at IH 4 h, 8 h, and 12 h. **(D)** The number of TIFs per cell are counted and plotted (*n* = 3). *p < 0.05, ***p < 0.001, ****p < 0.0001.

To model telomere-damaged neuronal cells, PC12 cells were infected with the Cas-Tel virus. Telomeric DNA damage was assessed by quantifying telomere dysfunction-induced foci (TIFs), defined as the colocalization of the DNA damage marker 53BP1 with telomeric regions. TIFs were identified by immunofluorescence, where 53BP1 (red fluorescence) overlapped with TelC-488-labeled telomere probes (green fluorescence) (Figure 3A). Immunofluorescence (IF) analysis revealed that the Cas-Tel group exhibited approximately a twofold increase in TIFs per cell compared to the negative control (NC) group (Figure 3B), confirming that Cas-Tel viral infection effectively induced telomere damage in PC12 cells.

**Figure 4:**
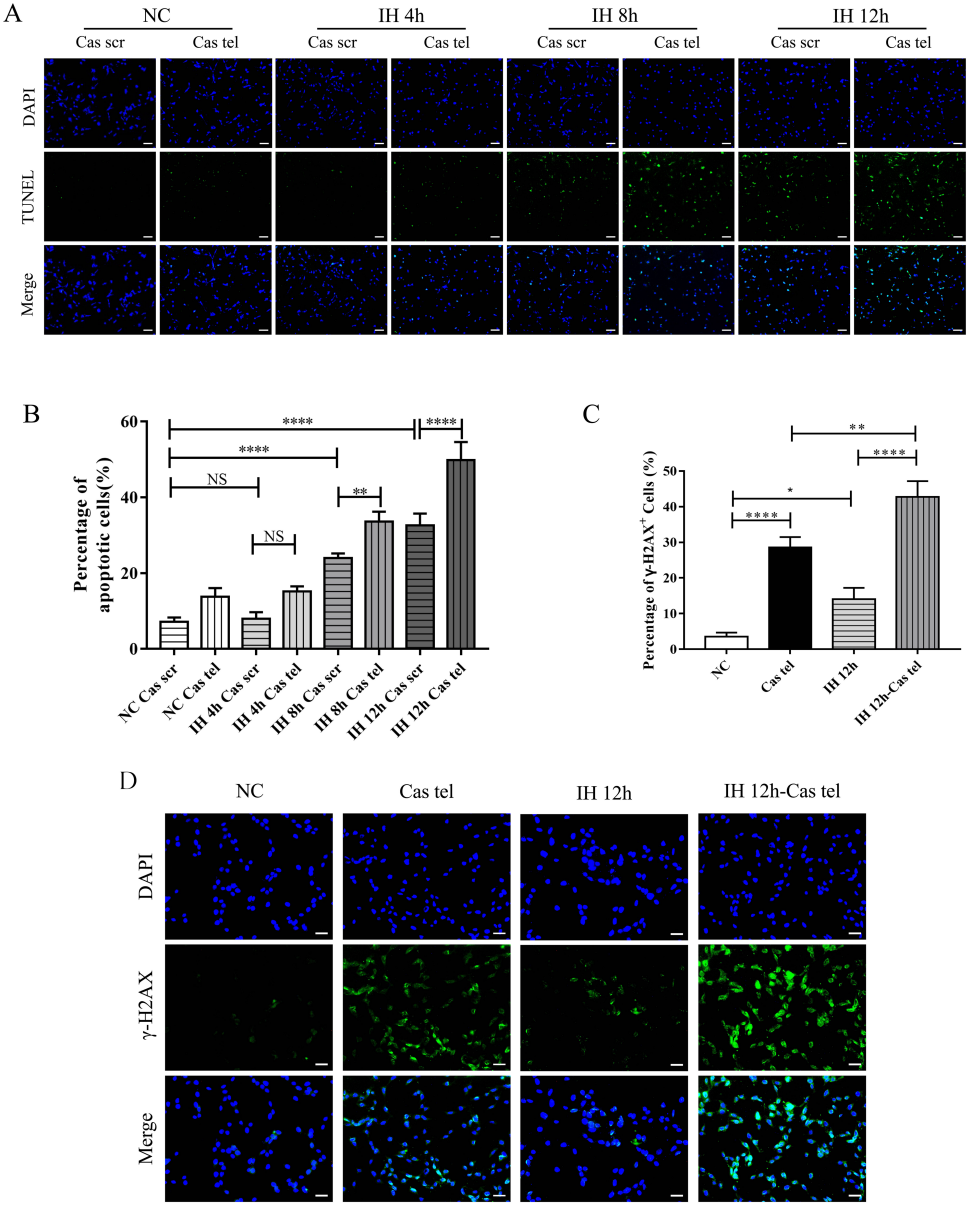
Telomere-damaged PC12 cells exacerbated IH-induced apoptosis and DNA damage. **(A,B)** Cell apoptosis was assessed by TUNEL (*n* = 3). Scale bars: 100 μm. **(C)** The number of *γ*-H2AX-positive cells represented as a percentage of total counted cells (*n* = 3). **(D)** Representative γ-H2AX staining in cells. Scale bars: 100 μm. *p < 0.05, **p < 0.01, ****p < 0.0001.

To examine the response of these cells to IH, each group of cells was exposed to IH for 4, 8, or 12 h. IF results demonstrated that IH exposure for 8 and 12 h significantly increased TIFs in a time-dependent manner (Figures 3C,D). Correspondingly, TUNEL staining revealed that IH exposure for 8 or 12 h significantly increased the rate of apoptosis, with 12 h inducing the most pronounced effect (Figures 4A,B). Based on these findings, and to establish a more robust experimental model, 12-h IH exposure was selected for subsequent experiments.

**Figure 5:**
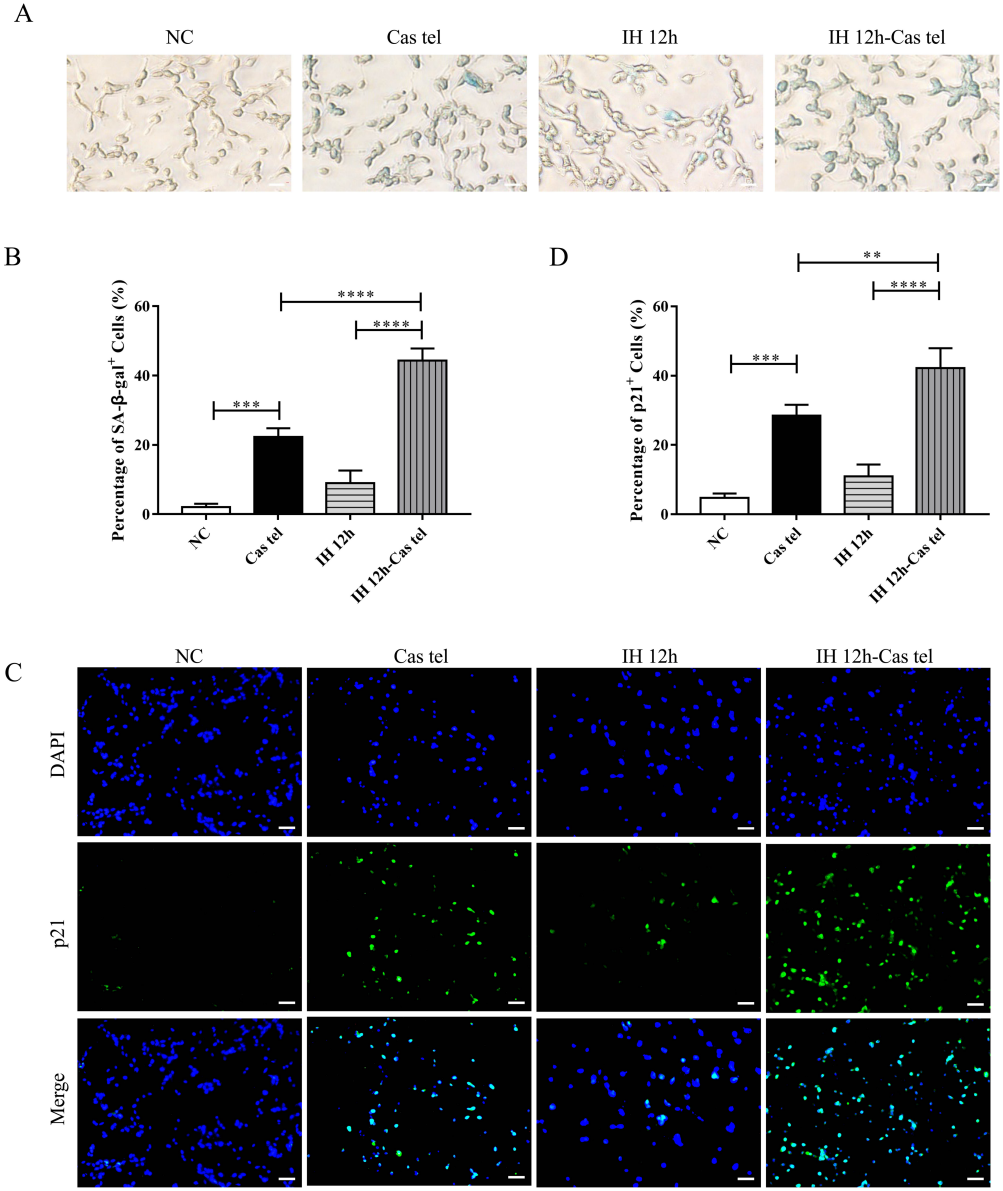
Telomere-damaged PC12 cells exacerbated IH-induced senescence-associated markers. **(A)** Representative SA-*β*-gal staining in cells. Scale bars: 100 μm. **(B)** The number of SA-β-gal-positive cells represented as a percentage of total counted cells (*n* = 3). **(C)** Representative p21 staining in cells. Scale bars: 100 μm. **(D)** The number of p21-positive cells represented as a percentage of total counted cells (*n* = 3). ^**^p < 0.01, ^***^p < 0.001, ****p < 0.0001.

To assess cellular stress responses, we analyzed DNA damage and senescence-associated markers. The percentage of *γ*-H2AX-positive was significantly higher in Cas-Tel group and was further increased upon IH exposure (Figures 4C,D). Consistently, both the percentage of SA-*β*-gal-positive cells and p21-positive cells showed a concordant upregulation (Figures 5A–D). In summary, telomere-damaged PC12 cells displayed heightened stress responses to IH, manifested by increased TIFs, apoptosis, and the upregulation of senescence- and DNA damage-associated markers.

### Comparative RNA sequencing revealed inflammatory signature in telomere-damaged cells under IH

**Figure 6:**
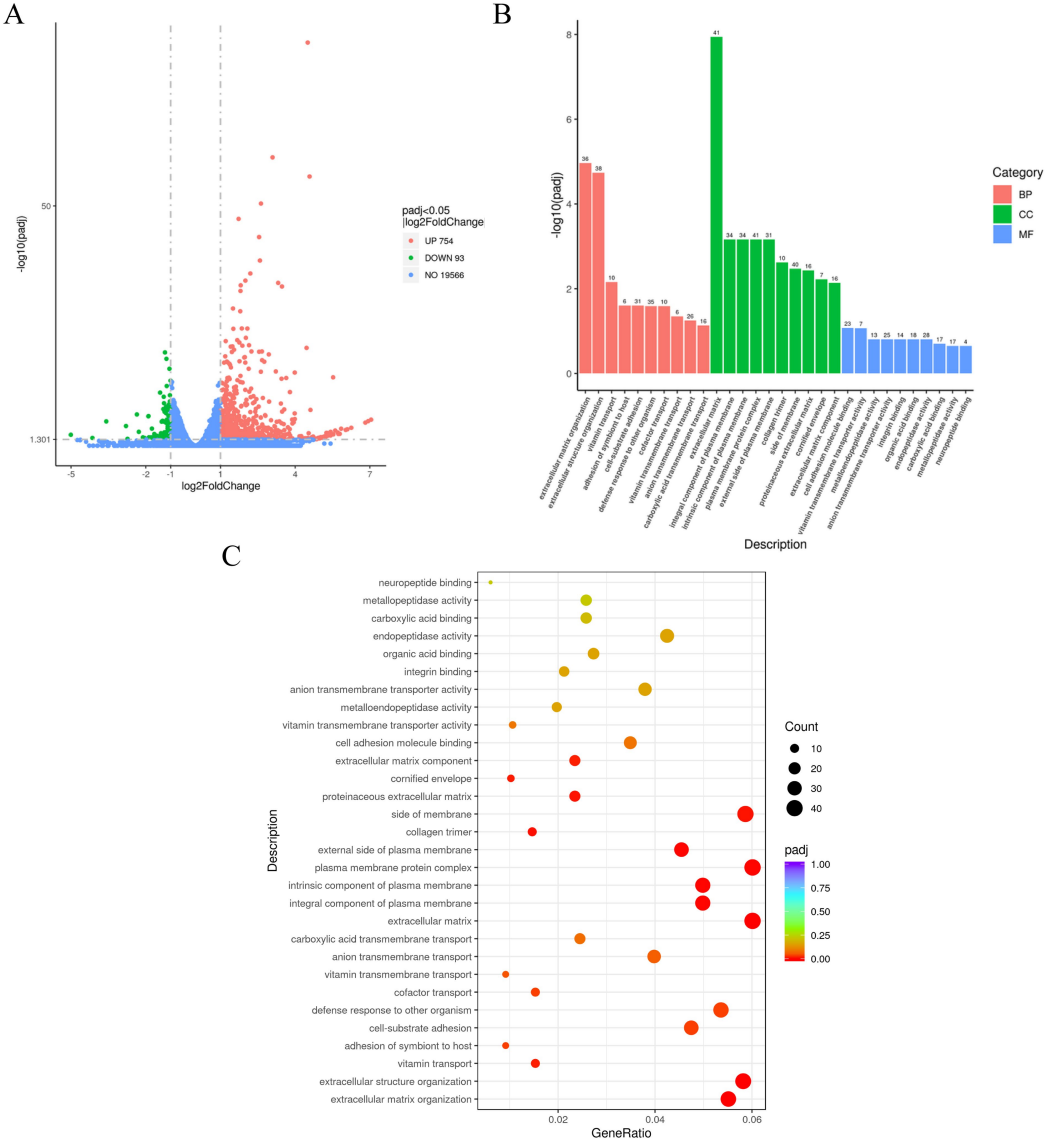
Differential gene expression and GO enrichment analysis. **(A)** Volcano map illustrated the DEGs between IH 12 h group and IH 12 h-Cas tel group. DEGs (highlighted in red for up-regulation and green for down-regulation) were defined as those with |log₂FC| > 1 and padj < 0.05. **(B)** GO analysis showed functions enriched by the upregulated differential genes in the IH 12 h-Cas tel group compared to the IH 12 h group. **(C)** The bubble chart illustrated the top TOP20 enriched functions in GO analysis.

To explore the molecular alterations associated with increased IH stress in telomere-damaged cells, we performed RNA sequencing on PC12 cells from four groups: NC, CasTel, IH 12 h, and IH 12 h–CasTel. Comparative transcriptomic analysis identified 847 differentially expressed genes (DEGs) in the IH 12 h–CasTel group compared to the IH 12 h group, including 754 upregulated and 93 downregulated genes (Figure 6A). Gene Ontology (GO) enrichment analysis of biological processes revealed that these DEGs were significantly associated with key functional categories, particularly extracellular matrix (ECM) organization, extracellular structure organization, cofactor transport, and cell–substrate adhesion (Figures 6B,C).

**Figure 7:**
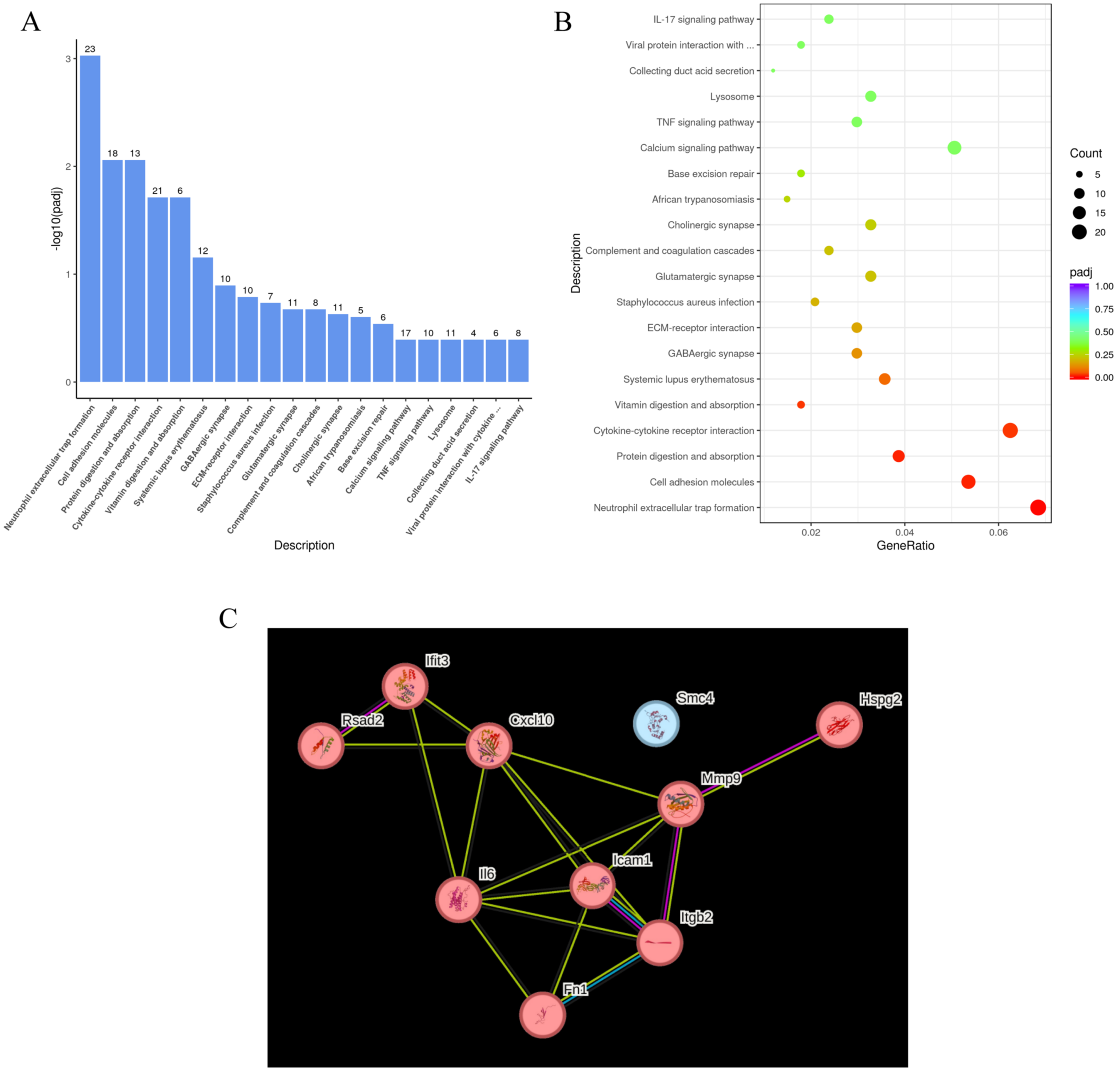
KEGG pathway enrichment analysis and PPI network analysis. **(A)** KEGG analysis showed pathways enriched by differential genes between IH 12 h-Cas tel group and IH 12 h group. **(B)** The bubble chart illustrated the top 20 enriched pathways in KEGG analysis. **(C)** PPI networks analysis showed top 10 protein node of differential genes between IH 12 h-Cas tel group and IH 12 h group.

Kyoto Encyclopedia of Genes and Genomes (KEGG) pathway analysis further showed that these DEGs were enriched in several biologically relevant pathways, including neutrophil extracellular trap formation, cell adhesion molecules, cytokine–cytokine receptor interactions, and ECM–receptor interactions. Additionally, key neural signaling pathways—such as GABAergic and glutamatergic synapses—and inflammatory pathways, including TNF and IL-17 signaling, were also significantly represented (Figures 7A,B).

Protein–protein interaction (PPI) network analysis of the DEGs identified the top 10 hub genes (Figure 7C). With the exception of Structural Maintenance of Chromosomes 4 (SMC4), the remaining nine genes clustered within a single functional module and were upregulated. Most of these hub genes were involved in immune-inflammatory responses, notably Interleukin-6 (IL-6), C-X-C motif chemokine ligand 10 (CXCL10), and Intercellular Cell Adhesion Molecule 1 (ICAM1). In summary, RNA sequencing of telomere-damaged PC12 cells exposed to IH revealed a distinct gene expression profile, characterized by the upregulation of pathways and hub genes central to immune and inflammatory responses.

### Rapamycin attenuated cellular stress in telomere-damaged cells under IH

**Figure 8:**
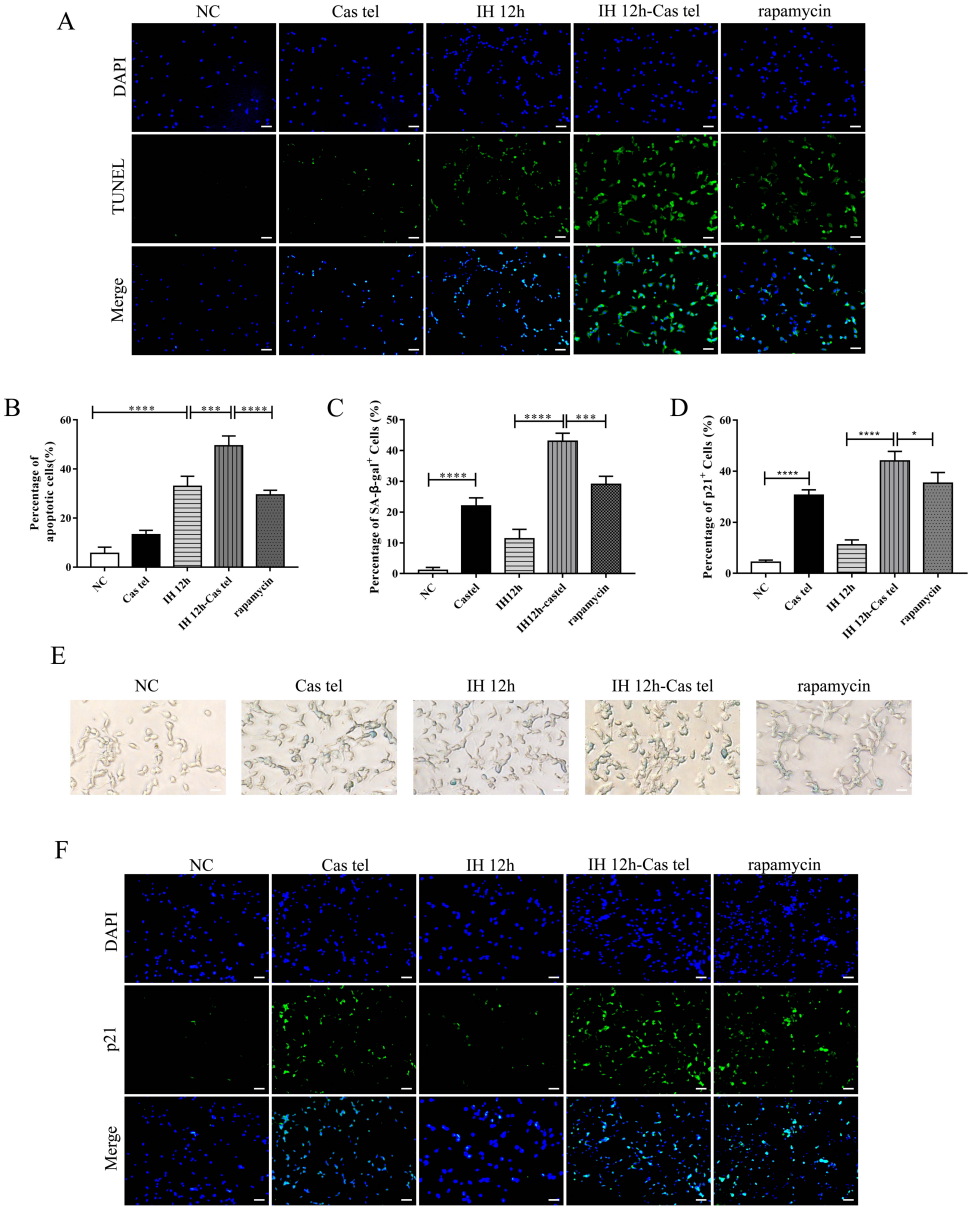
Rapamycin attenuated cellular stress in telomere-damaged cells under IH. **(A,B)** Cell apoptosis was assessed by TUNEL (*n* = 3). Scale bars: 100 μm. **(C)** The number of SA-β-gal-positive cells represented as a percentage of total counted cells (*n* = 3). **(D)** The number of p21-positive cells represented as a percentage of total counted cells (*n* = 3). **(E)** Representative SA-β-gal staining in cells. Scale bars: 100 μm. (F) Representative p21 staining in cells. Scale bars: 100 μm. Scale bars: 100 μm. *p < 0.05, ***p < 0.001, ****p < 0.0001.

To explore whether pharmacological intervention could mitigate the stress response in our cellular model, we treated telomere-damaged PC12 cells exposed to IH with rapamycin, an mTOR inhibitor with reported anti-aging properties. The TUNEL assay showed that the significant increase in apoptotic cells in the IH 12 h-Cas Tel group was markedly attenuated by rapamycin treatment (Figures 8A,B). Meanwhile, rapamycin treatment also effectively decreased the percentage of cells positive for the senescence markers SA-*β*-gal and p21 in the IH-Cas Tel group (Figures 8C–F). In summary, rapamycin treatment reversed the IH-induced increase in senescence-associated markers and apoptosis in telomere-damaged PC12 cells.

## Discussion

This study indicates that telomere dysfunction is associated with more severe detrimental effects of IH, the hallmark pathological feature of OSA. This association was observed at multiple levels: IH-G3 Tert^−/−^ mice exhibited more severe cognitive deficits and hippocampal damage, while telomere-damaged PC12 cells showed a heightened cellular stress response. Notably, intervention with agents targeting senescence-related pathways—the senolytic fisetin *in vivo* and the mTOR inhibitor Rapamycin *in vitro*—alleviated these pathological outcomes. Furthermore, RNA sequencing analysis identified that telomere damage under IH conditions may contribute to a distinct inflammatory transcriptional response.

We first employed G3 Tert^−/−^ progeroid mice, which at the experimental age of 6–8 weeks already displayed well-established core features of aging, including telomere attrition and elevated expression of senescence markers across multiple organs (Zhao et al., 2023). This model provides a robust platform for investigating accelerated aging-like pathologies and testing senotherapeutic interventions. Consistent behavioral and MRI analyses revealed that IH-G3 Tert^−/−^ mice developed more severe spatial memory deficits and hippocampal atrophy compared to WT controls, with the CA1, CA2, and DG subregions being particularly affected. The CA1 region is known to be highly sensitive to hypoxia, while the DG plays a crucial role in neurogenesis and episodic memory; both are common targets of impairment in OSA-related cognitive studies (Coimbra-Costa et al., 2017). Although CA3 is also involved in spatial memory, its relative resistance to hypoxic injury may explain the lack of volumetric loss observed in our study (Gozal et al., 2002; Klein et al., 2003). In our model, this specific pattern of regional susceptibility was markedly amplified in the context of accelerated aging. These findings indicate that the hippocampus of G3 Tert^−/−^ mice exhibits a region-specific and exacerbated susceptibility to IH-induced damage.

To validate the *in vivo* findings and dissect the cellular response mechanisms to IH, we established a telomere-damaged PC12 cell model. Initial time-course experiments identified 12-h IH exposure as a critical time point, concurrently inducing significant TIFs and apoptosis. Using this optimized model, we observed that telomere-damaged cells mounted a significantly heightened stress response to IH. This was evidenced by a concurrent increase in multiple markers—including SA-*β*-gal activity, p21, and *γ*-H2AX expression—alongside elevated apoptosis. However, whether a causal or temporal relationship exists between this co-occurring increase in senescence-associated phenotypes and apoptosis remains to be explored. Both outcomes may be driven in parallel by a common, severe upstream insult, or they may interact with each other. Elucidating the precise sequence and mechanism of this interaction will be an important objective for future research. The heightened stress response observed at the cellular level may contribute to the more severe hippocampal damage and cognitive decline observed in vivo, together suggesting an amplifying effect of telomere dysfunction on IH-induced injury.

To explore the molecular basis of the observed heightened stress response, we conducted RNA-Seq transcriptomic profiling on cells. Comparative transcriptomic analysis between IH-Cas Tel cells and IH-exposed controls revealed that the combined insult induced a distinct inflammatory transcriptional signature. DEGs were significantly enriched in gene sets related to immune-inflammatory signaling, including the TNF and IL-17 pathways, ECM remodeling, cofactor transport and cell-substrate adhesion. PPI network analysis identified IL-6, CXCL10, and ICAM-1 as central nodes, consistent with a pro-inflammatory transcriptional profile. The coordinated upregulation of these factors suggests that telomere dysfunction, in conjunction with IH, may facilitate a pro-inflammatory transcriptional state. Notably, the hub gene ICAM1—a cell adhesion molecule—is a well-established mediator of neuroinflammation (Li et al., 2022; Zhu et al., 2025). Its upregulation is associated with neuroinflammatory states and aging. Importantly, interventions targeting this class of adhesion molecules, such as vascular cell adhesion molecule 1 (VCAM1), have been shown to mitigate age-related brain changes, underscoring their potential therapeutic relevance (Yousef et al., 2019). Thus, this inflammatory transcriptional signature provides a key molecular correlate and a potential explanation for the exacerbated pathological outcomes observed in our models. Additionally, our transcriptomic data revealed significant enrichment in ECM-related pathways. ECM remodeling and ICAM-1 may play pivotal roles in neuroinflammation and neuronal integrity during aging. The ECM provides both structural scaffolding and critical signaling cues to neurons and undergoes age-dependent alterations that contribute to tissue dysfunction and impaired neural plasticity (Kurtz and Oh, 2012; Levi et al., 2020; Jo et al., 2021). In summary, the co-upregulation of inflammatory and ECM pathways raises the possibility of their interaction in forming a detrimental microenvironment that could exacerbate cellular stress. In this context, the specific functional roles of these key hub molecules warrant future validation using genetic or pharmacological approaches.

Given the observed association between telomere dysfunction and more severe IH-induced damage, we proceeded to explore whether interventions with anti-aging properties could confer protection. We assessed the senolytic agent fisetin *in vivo* and the mTOR inhibitor rapamycin *in vitro*. Fisetin, a polyphenolic flavonoid abundant in various fruits and vegetables, has emerged as a potent senolytic agent capable of selectively clearing senescent cells (Xing et al., 2023; Lee and Burns, 2024; Tavenier et al., 2024). Acute or intermittent administration of fisetin significantly attenuated senescence markers across multiple tissues in both progeroid and aged mice, aligning with a hit-and-run senolytic mechanism. When administered to wild-type mice late in life, fisetin treatment promoted the restoration of tissue homeostasis, attenuated age-related pathological manifestations, and extended both median and maximum lifespan (Yousefzadeh et al., 2018). In this study, fisetin ameliorated IH-induced cognitive decline, hippocampal atrophy, microstructural brain abnormalities, and neuronal apoptosis in G3 Tert^−/−^ mice. Rapamycin, an mTOR inhibitor, is widely recognized for its lifespan-extending and healthspan-promoting properties across diverse models (Harrison et al., 2009; Bjedov et al., 2010; Miller et al., 2011; Chung et al., 2019; Selvarani et al., 2021; Lee and Burns, 2024). Its mechanism involves the inhibition of the mTOR pathway, which promotes autophagy and mitigates cellular senescence (Cayo et al., 2021; Regan et al., 2022). In our in vitro model, rapamycin significantly reduced IH-induced senescence and apoptosis in our telomere-damaged PC12 cells. Although these agents operate through distinct mechanisms, their protective effects provide pharmacological evidence that interventions targeting aging-related pathways may alleviate the nerve damage associated with the combination of telomere dysfunction and IH. However, these findings must be interpreted within their experimental context. Both fisetin and rapamycin are pleiotropic compounds with multiple cellular targets. While their efficacy in our models supports the involvement of aging-associated pathways, it does not establish cellular senescence as the singular or exclusive causal mechanism. Future studies employing more specific genetic or pharmacological tools to modulate senescence will be required to definitively dissect the causal mechanisms and precisely delineate the roles of fisetin and rapamycin in this context.

Limitations of this study should be acknowledged. First, it is crucial to emphasize that while the progeria mouse model serves as a valuable system for simulating aspects of physiological aging, it does not fully capture the intricate and multifactorial essence of natural aging. Although our work provides evidence for an association between telomere dysfunction and exacerbated IH-induced cognitive deficits as well as nerve damage, the extent to which this specific mechanistic interaction (potentially involving inflammatory pathways) is activated and plays a central role in the naturally aged hippocampus or in OSA patients requires direct validation in corresponding aged models and clinical samples. Second, while PC12 cells are a widely used neuronal model, they do not fully recapitulate the functional characteristics of mature central neurons, particularly those in the hippocampus or cortex (Seta et al., 2002a,b; Chen et al., 2014; Mei et al., 2018; Singh et al., 2019). Additionally, the senescence-associated markers used are not specific and may also indicate DNA damage response or cellular stress. Therefore, our model demonstrates a heightened senescence-like state, with the precise classification requiring validation via more specific biomarkers or lineage-tracing approaches. Finally, this study was conducted exclusively in female G3 Tert^−/−^ mice. Although this aligns with epidemiological evidence of a heightened susceptibility to OSA-related cognitive decline in women, it limits the generalizability of our findings both to male subjects and to the context of natural chronological aging (Braley et al., 2024).

Future studies should adopt a broader panel of senescence biomarkers, such as p16^INK4a^ and senescence-associated secretory phenotype (SASP) factors, to more comprehensively characterize the senescent phenotype in our models. Most critically, replicating the core finding of synergistic neurotoxicity in naturally aged wild-type models is essential to establish its relevance to chronological aging and human OSA. Meanwhile, efforts should be made to validate the roles of IL-6, CXCL10, and other candidate molecules using genetic knockdown or pharmacological inhibition. The development of humanized models, such as iPSC-derived neurons and brain organoids, may bridge the gap between rodent studies and clinical application. Longitudinal studies assessing the cumulative impact of chronic IH in aging populations on cognition and brain architecture are also warranted. Additionally, future studies could explore combinatorial therapies that integrate senolytics with targeted anti-inflammatory agents, which may offer a strategy to achieve synergistic protection in relevant models.

In summary, this study links telomere dysfunction to more severe IH-induced cognitive and neuronal damage, reveals an associated inflammatory transcriptomic profile, and provides proof-of-concept that targeting senescence-related pathways can be protective. These findings offer a novel conceptual framework and candidate targets for understanding and potentially mitigating aging-related susceptibility to OSA-associated cognitive decline.

## Data Availability

The original contributions presented in the study are included in the article/supplementary material, further inquiries can be directed to the corresponding author.

## References

[ref1] Ayalon L. Ancoli-Israel S. Drummond S. P. (2010). Obstructive sleep apnea and age: a double insult to brain function? Am. J. Respir. Crit. Care Med. 182, 413–419. doi: 10.1164/rccm.200912-1805OC, 20395556 PMC2921601

[ref2] Benjafield A. V. Ayas N. T. Eastwood P. R. Heinzer R. Ip M. S. M. Morrell M. J. . (2019). Estimation of the global prevalence and burden of obstructive sleep apnoea: a literature-based analysis. Lancet Respir. Med. 7, 687–698. doi: 10.1016/S2213-2600(19)30198-5, 31300334 PMC7007763

[ref3] Bjedov I. Toivonen J. M. Kerr F. Slack C. Jacobson J. Foley A. . (2010). Mechanisms of life span extension by rapamycin in the fruit fly *Drosophila melanogaster*. Cell Metab. 11, 35–46. doi: 10.1016/j.cmet.2009.11.010, 20074526 PMC2824086

[ref4] Bombois S. Derambure P. Pasquier F. Monaca C. (2010). Sleep disorders in aging and dementia. J. Nutr. Health Aging 14, 212–217. doi: 10.1007/s12603-010-0052-7, 20191256 PMC12878873

[ref5] Braley T. J. Lyu X. Dunietz G. L. Schulz P. C. Bove R. Chervin R. D. . (2024). Sex-specific dementia risk in known or suspected obstructive sleep apnea: a 10-year longitudinal population-based study. Sleep Adv. 5:zpae077. doi: 10.1093/sleepadvances/zpae077, 39554998 PMC11568356

[ref6] Castriotta R. J. Murthy J. N. (2011). Sleep disorders in patients with traumatic brain injury: a review. CNS Drugs 25, 175–185. doi: 10.2165/11584870-000000000-00000, 21062105

[ref7] Cayo A. Segovia R. Venturini W. Moore-Carrasco R. Valenzuela C. Brown N. (2021). mTOR activity and autophagy in senescent cells, a complex partnership. Int. J. Mol. Sci. 22:8149. doi: 10.3390/ijms22158149, 34360912 PMC8347619

[ref8] Chang J. L. Goldberg A. N. Alt J. A. Mohammed A. Ashbrook L. Auckley D. . (2023). International consensus statement on obstructive sleep apnea. Int. Forum Allergy Rhinol. 13, 1061–1482. doi: 10.1002/alr.23079, 36068685 PMC10359192

[ref9] Chen T. I. Chiu H. W. Pan Y. C. Hsu S. T. Lin J. H. Yang K. T. (2014). Intermittent hypoxia-induced protein phosphatase 2A activation reduces PC12 cell proliferation and differentiation. J. Biomed. Sci. 21:46. doi: 10.1186/1423-0127-21-46, 24885237 PMC4058715

[ref10] Cheng M. Ye C. Tian C. Zhao D. Li H. Sun Z. . (2023). Engineered macrophage-biomimetic versatile nanoantidotes for inflammation-targeted therapy against Alzheimer's disease by neurotoxin neutralization and immune recognition suppression. Bioact. Mater. 26, 337–352. doi: 10.1016/j.bioactmat.2023.03.004, 36950153 PMC10027514

[ref11] Chung C. L. Lawrence I. Hoffman M. Elgindi D. Nadhan K. Potnis M. . (2019). Topical rapamycin reduces markers of senescence and aging in human skin: an exploratory, prospective, randomized trial. Geroscience 41, 861–869. doi: 10.1007/s11357-019-00113-y, 31761958 PMC6925069

[ref12] Coimbra-Costa D. Alva N. Duran M. Carbonell T. Rama R. (2017). Oxidative stress and apoptosis after acute respiratory hypoxia and reoxygenation in rat brain. Redox Biol. 12, 216–225. doi: 10.1016/j.redox.2017.02.014, 28259102 PMC5334548

[ref13] Cosentino F. I. Bosco P. Drago V. Prestianni G. Lanuzza B. Iero I. . (2008). The APOE epsilon4 allele increases the risk of impaired spatial working memory in obstructive sleep apnea. Sleep Med. 9, 831–839. doi: 10.1016/j.sleep.2007.10.015, 18083630

[ref14] Deegan P. C. McNicholas W. T. (1995). Pathophysiology of obstructive sleep apnoea. Eur. Respir. J. 8, 1161–1178. doi: 10.1183/09031936.95.08071161, 7589402

[ref15] Drager L. F. Togeiro S. M. Polotsky V. Y. Lorenzi-Filho G. (2013). Obstructive sleep apnea: a cardiometabolic risk in obesity and the metabolic syndrome. J. Am. Coll. Cardiol. 62, 569–576. doi: 10.1016/j.jacc.2013.05.045, 23770180 PMC4461232

[ref16] Driver A. S. Kodavanti P. R. Mundy W. R. (2000). Age-related changes in reactive oxygen species production in rat brain homogenates. Neurotoxicol. Teratol. 22, 175–181. doi: 10.1016/s0892-0362(99)00069-0, 10758346

[ref17] Gileles-Hillel A. Kheirandish-Gozal L. Gozal D. (2016). Biological plausibility linking sleep apnoea and metabolic dysfunction. Nat. Rev. Endocrinol. 12, 290–298. doi: 10.1038/nrendo.2016.22 26939978

[ref18] Gozal D. Capdevila O. S. Kheirandish-Gozal L. Crabtree V. M. (2007). APOE epsilon 4 allele, cognitive dysfunction, and obstructive sleep apnea in children. Neurology 69, 243–249. doi: 10.1212/01.wnl.0000265818.88703.83, 17636061

[ref19] Gozal E. Gozal D. Pierce W. M. Thongboonkerd V. Scherzer J. A. Sachleben L. R. Jr. . (2002). Proteomic analysis of CA1 and CA3 regions of rat hippocampus and differential susceptibility to intermittent hypoxia. J. Neurochem. 83, 331–345. doi: 10.1046/j.1471-4159.2002.01134.x, 12423243

[ref20] Guo Y. Miao Y. Wang K. Tan J. Jiao Z. Zhang Q. (2025). Different types of Nano lipid bubbles for protecting nerve cells from intermittent hypoxia damage. Tohoku J. Exp. Med. 267, 151–162. doi: 10.1620/tjem.2025.J008, 39880647

[ref21] Harrison D. E. Strong R. Sharp Z. D. Nelson J. F. Astle C. M. Flurkey K. . (2009). Rapamycin fed late in life extends lifespan in genetically heterogeneous mice. Nature 460, 392–395. doi: 10.1038/nature08221, 19587680 PMC2786175

[ref22] Hartleben B. Godel M. Meyer-Schwesinger C. Liu S. Ulrich T. Kobler S. . (2010). Autophagy influences glomerular disease susceptibility and maintains podocyte homeostasis in aging mice. J. Clin. Invest. 120, 1084–1096. doi: 10.1172/JCI39492 20200449, 20200449 PMC2846040

[ref23] Huang X. Zhang Z. Lan X. Song X. Dong Y. Jia S. . (2025). The association between hypoxic burden and the risk of cognitive impairment in patients with obstructive sleep apnea. Sleep 48:zsae269. doi: 10.1093/sleep/zsae269, 39570770 PMC11893536

[ref24] Jo Y. Hwang S. H. Jang J. (2021). Employing extracellular matrix-based tissue engineering strategies for age-dependent tissue degenerations. Int. J. Mol. Sci. 22:9367. doi: 10.3390/ijms22179367, 34502277 PMC8431718

[ref25] Klein J. B. Gozal D. Pierce W. M. Thongboonkerd V. Scherzer J. A. Sachleben L. R. . (2003). Proteomic identification of a novel protein regulated in CA1 and CA3 hippocampal regions during intermittent hypoxia. Respir. Physiol. Neurobiol. 136, 91–103. doi: 10.1016/s1569-9048(03)00074-0, 12853002

[ref26] Kurtz A. Oh S. J. (2012). Age related changes of the extracellular matrix and stem cell maintenance. Prev. Med. 54 Suppl, S50–S56. doi: 10.1016/j.ypmed.2012.01.003, 22285947

[ref27] Lal C. Strange C. Bachman D. (2012). Neurocognitive impairment in obstructive sleep apnea. Chest 141, 1601–1610. doi: 10.1378/chest.11-2214, 22670023

[ref28] Lee E. Burns M. (2024). The effects of Fisetin on reducing biological aging: a pilot study. Altern. Ther. Health Med. 30, 6–10. Available online at: https://alternative-therapies.com/abstract/index.html?id=161254 39269340

[ref29] Leng Y. McEvoy C. T. Allen I. E. Yaffe K. (2017). Association of Sleep-Disordered Breathing with Cognitive Function and Risk of cognitive impairment: a systematic review and Meta-analysis. JAMA Neurol. 74, 1237–1245. doi: 10.1001/jamaneurol.2017.2180, 28846764 PMC5710301

[ref30] Levi N. Papismadov N. Solomonov I. Sagi I. Krizhanovsky V. (2020). The ECM path of senescence in aging: components and modifiers. FEBS J. 287, 2636–2646. doi: 10.1111/febs.15282, 32145148

[ref31] Levy P. Kohler M. McNicholas W. T. Barbe F. McEvoy R. D. Somers V. K. . (2015). Obstructive sleep apnoea syndrome. Nat. Rev. Dis. Primers 1:15015. doi: 10.1038/nrdp.2015.15, 27188535

[ref32] Li C. Li Q. Liu S. Li J. Yu W. Li Y. . (2022). sVCAM1 in the Hippocampus contributes to postoperative cognitive dysfunction in mice by inducing microglial activation through the VLA-4 receptor. Mol. Neurobiol. 59, 5485–5503. doi: 10.1007/s12035-022-02924-1, 35727436

[ref33] McNicholas W. T. Pevernagie D. (2022). Obstructive sleep apnea: transition from pathophysiology to an integrative disease model. J. Sleep Res. 31:e13616. doi: 10.1111/jsr.13616, 35609941 PMC9539471

[ref34] Mei H. F. Poonit N. Zhang Y. C. Ye C. Y. Cai H. L. Yu C. Y. . (2018). Activating adenosine A1 receptor accelerates PC12 cell injury via ADORA1/PKC/KATP pathway after intermittent hypoxia exposure. Mol. Cell. Biochem. 446, 161–170. doi: 10.1007/s11010-018-3283-2, 29380238

[ref35] Miller R. A. Harrison D. E. Astle C. M. Baur J. A. Boyd A. R. de Cabo R. . (2011). Rapamycin, but not resveratrol or simvastatin, extends life span of genetically heterogeneous mice. J. Gerontol. A Biol. Sci. Med. Sci. 66, 191–201. doi: 10.1093/gerona/glq178, 20974732 PMC3021372

[ref36] Regan J. C. Lu Y. X. Urena E. Meilenbrock R. L. Catterson J. H. Kissler D. . (2022). Sexual identity of enterocytes regulates autophagy to determine intestinal health, lifespan and responses to rapamycin. Nat. Aging 2, 1145–1158. doi: 10.1038/s43587-022-00308-7, 37118538 PMC10154239

[ref37] Salminen L. E. Paul R. H. (2014). Oxidative stress and genetic markers of suboptimal antioxidant defense in the aging brain: a theoretical review. Rev. Neurosci. 25, 805–819. doi: 10.1515/revneuro-2014-0046, 25153586 PMC6378111

[ref38] Selvarani R. Mohammed S. Richardson A. (2021). Effect of rapamycin on aging and age-related diseases-past and future. Geroscience 43, 1135–1158. doi: 10.1007/s11357-020-00274-1, 33037985 PMC8190242

[ref39] Seta K. Kim H. W. Ferguson T. Kim R. Pathrose P. Yuan Y. . (2002a). Genomic and physiological analysis of oxygen sensitivity and hypoxia tolerance in PC12 cells. Ann. N. Y. Acad. Sci. 971, 379–388. doi: 10.1111/j.1749-6632.2002.tb04500.x, 12438156

[ref40] Seta K. A. Spicer Z. Yuan Y. Lu G. Millhorn D. E. (2002b). Responding to hypoxia: lessons from a model cell line. Sci. STKE 2002:re11. doi: 10.1126/stke.2002.146.re11, 12189251

[ref41] Singh B. L. Chen L. Cai H. Shi H. Wang Y. Yu C. . (2019). Activation of adenosine A2a receptor accelerates and A2a receptor antagonist reduces intermittent hypoxia induced PC12 cell injury via PKC-KATP pathway. Brain Res. Bull. 150, 118–126. doi: 10.1016/j.brainresbull.2019.05.015, 31129168

[ref42] Sun Y. Miao Y. Dai S. Wang K. Tan J. Jiao Z. . (2026). The use of xenon magnetic lipid bubbles to detect and treat intermittent hypoxia-induced cognitive impairment. J. Control. Release 389:114478. doi: 10.1016/j.jconrel.2025.114478, 41319964

[ref43] Tavenier J. Nehlin J. O. Houlind M. B. Rasmussen L. J. Tchkonia T. Kirkland J. L. . (2024). Fisetin as a senotherapeutic agent: evidence and perspectives for age-related diseases. Mech. Ageing Dev. 222:111995. doi: 10.1016/j.mad.2024.111995, 39384074

[ref44] Tian Q. Sun J. Li X. Liu J. Zhou H. Deng J. . (2024). Association between sleep apnoea and risk of cognitive impairment and Alzheimer's disease: a meta-analysis of cohort-based studies. Sleep Breath. 28, 585–595. doi: 10.1007/s11325-023-02934-w, 37857768

[ref45] Venkateshappa C. Harish G. Mahadevan A. Srinivas Bharath M. M. Shankar S. K. (2012). Elevated oxidative stress and decreased antioxidant function in the human hippocampus and frontal cortex with increasing age: implications for neurodegeneration in Alzheimer's disease. Neurochem. Res. 37, 1601–1614. doi: 10.1007/s11064-012-0755-8, 22461064

[ref46] Wang C. G. Ma W. H. Liu R. Yang M. Y. Yang Y. Ding Y. L. (2022). The effect of continuous adductor canal block combined with distal interspace between the popliteal artery and capsule of the posterior knee block for total knee arthroplasty: a randomized, double-blind, controlled trial. BMC Anesthesiol. 22:175. doi: 10.1186/s12871-022-01712-7, 35668348 PMC9169338

[ref47] Wilde M. C. Castriotta R. J. Lai J. M. Atanasov S. Masel B. E. Kuna S. T. (2007). Cognitive impairment in patients with traumatic brain injury and obstructive sleep apnea. Arch. Phys. Med. Rehabil. 88, 1284–1288. doi: 10.1016/j.apmr.2007.07.012, 17908570

[ref48] Xing X. Liang Y. Li Y. Zhao Y. Zhang Y. Li Z. . (2023). Fisetin delays postovulatory oocyte aging by regulating oxidative stress and mitochondrial function through Sirt1 pathway. Molecules 28:5533. doi: 10.3390/molecules28145533, 37513404 PMC10384696

[ref49] Yaffe K. Falvey C. M. Hoang T. (2014). Connections between sleep and cognition in older adults. Lancet Neurol. 13, 1017–1028. doi: 10.1016/S1474-4422(14)70172-3, 25231524

[ref50] Yaffe K. Laffan A. M. Harrison S. L. Redline S. Spira A. P. Ensrud K. E. . (2011). Sleep-disordered breathing, hypoxia, and risk of mild cognitive impairment and dementia in older women. JAMA 306, 613–619. doi: 10.1001/jama.2011.1115, 21828324 PMC3600944

[ref51] Yeghiazarians Y. Jneid H. Tietjens J. R. Redline S. Brown D. L. El-Sherif N. . (2021). Obstructive sleep apnea and cardiovascular disease: a scientific statement from the American Heart Association. Circulation 144, e56–e67. doi: 10.1161/CIR.0000000000000988, 34148375

[ref52] Yousef H. Czupalla C. J. Lee D. Chen M. B. Burke A. N. Zera K. A. . (2019). Aged blood impairs hippocampal neural precursor activity and activates microglia via brain endothelial cell VCAM1. Nat. Med. 25, 988–1000. doi: 10.1038/s41591-019-0440-4, 31086348 PMC6642642

[ref53] Yousefzadeh M. J. Zhu Y. McGowan S. J. Angelini L. Fuhrmann-Stroissnigg H. Xu M. . (2018). Fisetin is a senotherapeutic that extends health and lifespan. EBioMedicine 36, 18–28. doi: 10.1016/j.ebiom.2018.09.015, 30279143 PMC6197652

[ref54] Zhang Q. Gao W. Y. Zhang Y. Chen B. Y. Chen Z. Zhang W. S. . (2013). Protective effects of astragalus extract against intermittent hypoxia-induced hippocampal neurons impairment in rats. Chin. Med. J. 126, 1551–1554. Available online at: https://journals.lww.com/cmj/fulltext/2013/04200/protective_effects_of_astragalus_extract_against.27.aspx 23595393

[ref55] Zhang Y. Miao Y. Xiong X. Tan J. Han Z. Chen F. . (2023). Microglial exosomes alleviate intermittent hypoxia-induced cognitive deficits by suppressing NLRP3 inflammasome. Biol. Direct 18:29. doi: 10.1186/s13062-023-00387-5, 37312196 PMC10262550

[ref56] Zhao R. Kou H. Jiang D. Wang F. (2023). Exploring the anti-aging effects of fisetin in telomerase-deficient progeria mouse model. PeerJ 11:e16463. doi: 10.7717/peerj.16463, 38107570 PMC10722989

[ref57] Zhu S. Zhang Y. Li C. Deng Z. Yin Y. Dong Z. . (2025). Multiple synergistic anti-aging effects of vascular cell adhesion molecule 1 functionalized nanoplatform to improve age-related neurodegenerative diseases. J. Control. Release 379, 363–376. doi: 10.1016/j.jconrel.2025.01.022, 39798706

